# Positive emotion impedes emotional but not cognitive conflict processing

**DOI:** 10.3758/s13415-017-0504-1

**Published:** 2017-03-20

**Authors:** Artyom Zinchenko, Christian Obermeier, Philipp Kanske, Erich Schröger, Sonja A. Kotz

**Affiliations:** 1 International Max Planck Research School on Neuroscience of Communication (IMPRS NeuroCom), Leipzig, Germany; 2 Department of Neuropsychology, Max Planck Institute for Human Cognitive and Brain Sciences, 04103 Leipzig, Germany; 3 Department of Social Neuroscience, Max Planck Institute for Human Cognitive and Brain Sciences, Leipzig, Germany; 4 Institute of Psychology, University of Leipzig, Leipzig, Germany; 5 Faculty of Psychology and Neuroscience, Department of Neuropsychology and Psychopharmacology, Maastricht University, Maastricht, The Netherlands

**Keywords:** Positive emotion, Cognitive control, Cognitive conflict, Emotional conflict

## Abstract

Cognitive control enables successful goal-directed behavior by resolving a conflict between opposing action tendencies, while emotional control arises as a consequence of emotional conflict processing such as in irony. While negative emotion facilitates both cognitive and emotional conflict processing, it is unclear how emotional conflict processing is affected by positive emotion (e.g., humor). In 2 EEG experiments, we investigated the role of positive audiovisual target stimuli in cognitive and emotional conflict processing. Participants categorized either spoken vowels (cognitive task) or their emotional valence (emotional task) and ignored the visual stimulus dimension. Behaviorally, a positive target showed no influence on cognitive conflict processing, but impeded emotional conflict processing. In the emotional task, response time conflict costs were higher for positive than for neutral targets. In the EEG, we observed an interaction of emotion by congruence in the P200 and N200 ERP components in emotional but not in cognitive conflict processing. In the emotional conflict task, the P200 and N200 conflict effect was larger for emotional than neutral targets. Thus, our results show that emotion affects conflict processing differently as a function of conflict type and emotional valence. This suggests that there are conflict- and valence-specific mechanisms modulating executive control.

**Electronic supplementary material:**

The online version of this article (doi:10.3758/s13415-017-0504-1) contains supplementary material, which is available to authorized users.

The ability to integrate distinct sensory and specifically emotional input into a coherent percept is one of the major challenges affecting social interaction as it allows us to understand another person’s intentions or emotional state. Frequently, multimodal emotional information expressed by a social partner is ambiguous and, consequently, requires us to handle and interpret such conflicting information in effective communication (e.g., irony or humor; Pexman, 2008; Watanabe et al., 2014). For instance, irony often relies on multimodal expressions where emotional information expressed by the face and voice are in conflict (Wang, Lee, Sigman, & Dapretto, 2007). Thus*, emotional conflict* is created between stimuli of different emotional valence (e.g., neutral face and angry voice). Emotional conflict processing is known to produce longer reaction times (RTs) and higher error rates (Egner & Hirsch, 2005; Zinchenko, Kanske, Obermeier, Schröger, & Kotz, 2015). It seems to reflect increased competition for limited attentional resources and requires inhibition of emotional distractors (Etkin, Egner, Peraza, Kandel, & Hirsch, 2006).

Previous studies have mostly concentrated on cognitive conflict processing, which arises between opposing information sources (e.g., Stroop task). It has been shown that cognitive conflict processing can be influenced by the emotional quality of a target stimulus (Kanske, 2012; Xue et al., 2013). Recent data extend this effect to emotional conflict processing (Zinchenko et al., 2015). Participants watched short congruent and incongruent audiovisual videos and were asked to categorize spoken vowels (A/O; cognitive task) or their emotional valence (neutral/negative; emotional task). In other words, in the cognitive task the target was either neutral or emotional, but the emotional quality of the target was task irrelevant, while in the emotional task, the emotional quality of the target was task relevant (negative or neutral). The results indicated that negative targets lead to faster responses in both the cognitive and emotional tasks and to a conflict-specific modulation of the N100 component of the event-related potential (ERP). It is not clear, however, whether a positive target would lead to similar effects in cognitive and emotional conflict processing. There is some evidence that positive and negative emotions represent complementary, but distinct neuropsychological functions (Fredrickson, 1998, 2001). For instance, although both positive and negative emotions are salient stimuli that attract attention (Pilarczyk & Kuniecki, 2014), negative emotions seem to narrow one’s breadth of attention (Eysenck et al., 2007; Fredrickson, 2001), while positive emotions tend to broaden an individual’s scope of visual attention (e.g., Johnson et al., 2010). Furthermore, positive emotional conflict processing represents, among others, an important and adaptive social function (e.g., understanding humor). Therefore, the current study was designed to probe the influence of positive emotion on cognitive and emotional conflict processing.

There is evidence that positive emotions, similarly to negative emotions, speed up cognitive conflict processing when a target is emotional (Kanske & Kotz, 2011b), and modulate the conflict-sensitive N200 component (Kanske & Kotz, 2011a). On the other hand, positive emotions are associated with greater Flanker interference (Rowe, Hirsh, & Anderson, 2007) and may also have detrimental effects on executive control when a nontarget stimulus (e.g., presented shortly before the target stimulus) is emotional (e.g., Blair, Smith, & Mitchell, 2007; Dreisbach, 2006). Finally, there is also some evidence that positive emotions have no influence on cognitive control (Martin & Kerns, 2011). Thus, there is mixed evidence regarding the influence of positive emotions on cognitive control. Furthermore, the role of a positive target in emotional conflict processing has not yet been addressed, as previous studies used mostly negative emotions and/or stimuli where both target and nontarget stimuli were emotional (Egner, Etkin, Gale, & Hirsch, 2008).

Similar to Zinchenko et al. (2015), we varied the source of conflict between nonemotional (cognitive task) and emotional (emotional task) stimulus dimensions with similar stimuli in both tasks. We measured ERPs to study and differentiate early conflict-specific cognitive-affective processes in both tasks (Luck, 2005). Further, the study used dynamic multisensory neutral and positive stimuli in order to maximize robust neural responses to stimuli used in social communication (e.g., Klasen, Chen, & Mathiak, 2012; Liu et al., 2012; Schröger & Widmann, 1998). In both experiments participants perceived emotions expressed in multisensory stimuli. Finally, the current set of experiments included neutral control stimuli to address the question of how the emotional quality of the target stimulus affects cognitive and emotional conflict processing. Therefore, the present set of experiments tested whether cognitive and emotional conflict processing is influenced by the emotional quality of the target stimulus.

Based on the mixed results of previous studies looking at positive emotion in conflict tasks, we aimed to test whether positive and neutral emotions would (a) reduce the RT conflict effect (Kanske & Kotz, 2012), (b) increase the RT conflict effect (Rowe et al., 2007), or whether (c) positive emotion would not affect executive control at all (Martin & Kerns, 2011). Positive emotions signal potential reward (food, sex) and are linked to the motivational approach system (Coupland et al., 2004; Kanske & Kotz, 2011). Furthermore, positive emotions mediate stress recovery (Ong, Bergeman, Bisconti, & Wallace, 2006), facilitate audiovisual binding (Kitamura, Watanabe, & Kitagawa 2015), and can even influence physical health (Kok et al., 2013). These effects of positive emotions ensure rapid motor and attentional reactions in potentially rewarding situations.

A number of studies showed that both positive and negative emotions modulate cognitive conflict processing by reducing the RT conflict effect (Kanske & Kotz, 2010, 2011). Interestingly, these studies found comparable modulation of ERP components for both positive and negative emotional stimuli in a cognitive conflict task. These findings imply that emotion has a comparable influence on cognitive control, irrespective of valence. Therefore, we hypothesized that positive emotions should modulate early attentional processing (e.g., attract attention) and should facilitate both cognitive and emotional conflict processing similarly to negative emotions (Zinchenko et al., 2015). Additionally, positive and negative emotions may also elicit and modulate comparable conflict-sensitive ERP components (i.e., N100, P200, and N200). Incongruent compared to congruent stimuli elicit an enhanced N200 response (Bruin & Wijers, 2002; Kopp, Mattler, Goertz, & Rist, 1996; van Veen & Carter, 2002). This effect is typically found over frontocentral electrode sites (Heil, Osman, Wiegelmann, Rolke, & Hennighausen, 2000; Kopp, Rist, & Mattler, 1996, Kanske & Kotz, 2011b), as well as at posterior electrode-sites (Hughes, Velmans, & De Fockert, 2009; Zinchenko et al., 2015). Furthermore, it was shown that positive emotions modulate the conflict-sensitive N200 ERP component (Kanske & Kotz, 2011b). Therefore, we expected to observe a larger N200 conflict effect for positive than for neutral stimuli in the cognitive task. Whether or not positive emotions would modulate the N200 in the emotional task was less clear as it has not been tested before. Some previous studies showed that negative emotions modulate the N200 conflict effect in an emotional task (Zinchenko et al., 2015), while other studies did not observe this effect (Alguacil, Tudela, & Ruz, 2013; Ruz, Madrid, & Tudela, 2013).

Additionally, multisensory stimuli modulate the N100 and P200 ERP responses, which are usually associated with early feature processing (Jessen & Kotz, 2011; Kokinous, Kotz, Tavano, & Schroger, 2014; Paulmann, Jessen, & Kotz, 2009). The N100 is influenced by attention (Hillyard, Hink, Schwent, & Picton, 1973), congruence (Atkinson, Drysdale, & Fulham, 2003), and emotion (Scott, O’Donnell, Leuthold, & Sereno, 2009), while the P200 is also modulated by emotion (Paulmann, Jessen, & Kotz 2009) and task-relevance (Michalski, 2000). Similarly to the N200, the conflict-related N100 and P200 components were observed either anteriorly (Ho, Schröger, & Kotz 2014; Kokinous et al., 2015; Liu et al., 2012) or posteriorly (Gerdes et al., 2013; Ho et al., 2015). Thus, we predicted a conflict-driven enhancement of earlier ERP components (i.e., N100, P200) compared to previous unimodal studies that mostly found conflict-driven activations in the N200 ERP component (e.g., Kanske & Kotz, 2011a). In line with our previous findings, we expected to observe a smaller P200 amplitude to incongruent than to congruent trials in both cognitive and emotional tasks with either anterior or posterior distribution (Kokinous et al., 2014; Zinchenko et al., 2015).

Finally, our previous study found a conflict-specific N100 dissociation (Zinchenko et al., 2015). The authors reported a larger conflict effect for emotional than for neutral trials in the cognitive task and a larger conflict effect for neutral than for emotional trials in the emotional task (Zinchenko et al., 2015). However, another study exploring cognitive and emotional conflict processing did not observe such a dissociation (Alguacil et al., 2013).

## Experiment 1

### Method

#### Participants

Twenty-four participants (12 female, mean age = 24.5 years, *SD* = 4), all right-handed (Edinburgh Handedness Inventory score *ME* = 89.3, *SD* = 13.2), with normal or corrected-to-normal vision and normal self-reported hearing, participated in the two experiments. All participants signed a written informed consent form and received payment for their participation. The experiment was conducted in accordance with the guidelines of the Declaration of Helsinki and approved by the Ethics Committee of the University of Leipzig.

#### Stimulus material

**Fig. 1:**
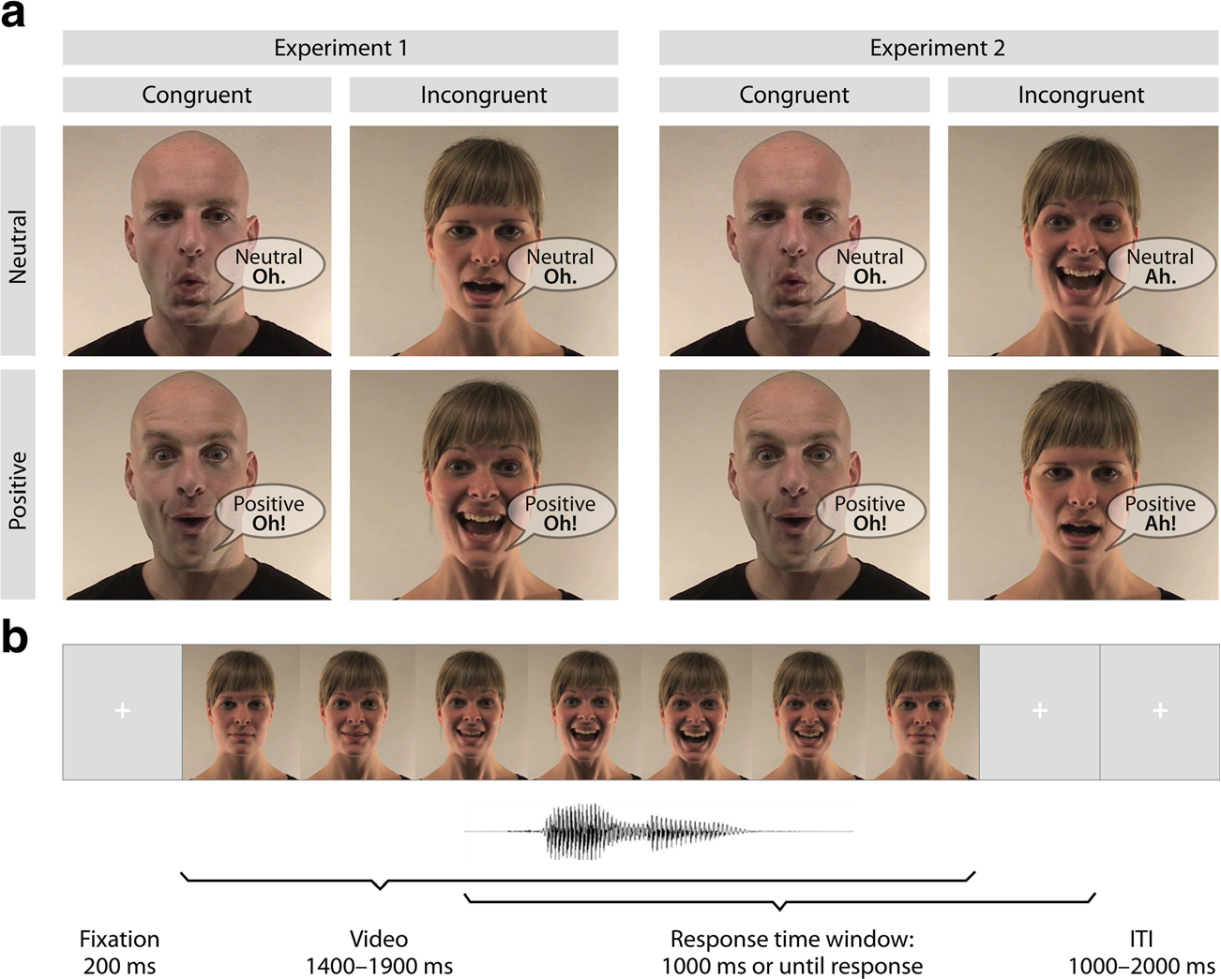
**a** Examples of stimuli in Experiments 1 and 2: The female and the male actors vocalized the interjections “A” and “O” in either a positive or neutral tone of voice. Incongruity was created by a mismatch of the vocalizations and the video components in Experiment 1 and mismatches in emotion of audio and video components in Experiment 2. **b** Example of a trial sequence

Stimuli consisted of short video clips of a male and a female semiprofessional actor pronouncing the interjections “A” and “O” in a neutral and a positive (i.e., happy) emotional tone of voice (see Fig. 1a). The facial expressions in the video started with a neutral face, evolved to peak expression, and returned back to a neutral expression. The sounds in all videos were normalized to 70 dB using root mean square (RMS) in Final Cut Pro. We created eight congruent and eight incongruent videos by matching or mismatching vocalizations of the face and voice (e.g., voice pronouncing “A” with facial lip movement corresponding to “A” vs. “O”, respectively). Videos were overlaid with the incongruent sound in Final Cut Pro 7 (Apple Inc.) using the onset of the original video sound for alignment. The emotional quality of the face and voice always matched in both congruent and incongruent conditions and could either be positive or neutral. The duration of the videos ranged from 1 to 2 seconds. There were no time differences between conditions before the audio onset, but the total video duration was longer for positive than for neutral stimuli. Additionally, we observed no differences between conditions with regard to the amount of movement, arousal, expressiveness, and emotion identification (see Supplementary Material for details).

Experiment 1 used a 2 (emotional, neutral) × 2 (congruent, incongruent) factorial design and was split into four blocks. Each block comprised 52 videos (26 emotional and 26 neutral, half were congruent and the other half incongruent) presented in a pseudorandomized order. Overall, there were 208 trials and testing took approximately 45 minutes per participant.

#### Procedure

Participants were seated in a dimly lit sound-attenuated booth about 1 meter from a computer screen. Sounds were delivered via headphones. Each trial started with a fixation cross on a blank computer screen for 200 ms (see Fig. 1b). Subsequently, a video was presented and played in full duration (i.e., video was not cut off after the response). Participants were instructed to report whether the vocalization of the voice was an “A” or “O,” irrespective of the facial expression that could be congruent or incongruent. To ensure that faces were not ignored, participants were sometimes (10% of all trials) asked a second question about which vowel the lip movement of the face expressed (i.e., “A” or “O”). These additional questions were presented randomly; the response time to these questions was not limited, and these trials were not used for further analysis (all participants answered >85% questions correctly and were included for further analysis). The response time window for the main question was 1,000 ms starting from voice onset. If participants did not respond within 1,000 ms, the words “try to respond faster” appeared on the screen for 200 ms. If participants answered incorrectly, the word “wrong” appeared on the screen. Button presses were counterbalanced across participants. An ITI of 1000, 1250, 1500, 1750, and 2000 ms was used randomly before the onset of the next trial.

Because we used very similar stimuli for the two types of conflicts, and to avoid carryover effects, we tested cognitive and emotional tasks on 2 separate days with at least 3 days between the two sessions. Also, we counterbalanced the order of the presentation of the two types of conflict: Half of participants performed the cognitive task first and the other half started with the emotional task.

##### EEG recording and preprocessing

EEG was recorded from 59 Ag/AgCl scalp electrodes (10-10 system). We used Brain Vision Recorder (Brain Products GmbH, Munich, Germany) and a BRAINAMP amplifier (DC to 250 Hz). The sampling rate was 500 Hz. The left mastoid served as a reference, and the sternum as ground. The vertical and horizontal electrooculogram was measured for artifact rejection purposes. The impedance was kept below 5 kΩ. EEG data were analyzed using the FieldTrip (V. 0.20120501) toolbox (Oostenveld, Fries, Maris, & Schoffelen, 2011) running on MATLAB 8.1 R2013a (The MathWorks, Natick, MA, USA). Electrodes were rereferenced offline to linked mastoids. Only correct trials were chosen for data processing. Extended epochs of 2,000 ms before and after the video onset were extracted. Epochs containing excessive muscle activity or jump artifacts were rejected. Data were band-pass filtered using a two-pass Butterworth IIR filter with a frequency pass band of 0.1 to 100 Hz (order of four). Independent component analysis (ICA) was conducted using the runica algorithm (Makeig et al., 1997). Subsequently, components were visually inspected and those showing ocular, muscle, heart, and electrode artifacts were manually rejected (mean number of components removed = 16.23, *SD* = 5.4). Subsequently, individual epochs were visually scanned, and epochs containing artifacts were manually discarded. Approximately 20% of trials (incorrect responses, artifacts) were excluded from the final statistical analysis.

##### Data analysis

For the statistical analysis, smaller epochs were selected (-200 to 1,000 ms time locked to the voice onset). Single-subject averages were calculated for each session and condition. Based on previous studies (Kanske & Kotz, 2010), four regions of interest were defined: the left anterior (FP1, AF3, AF7, F3, F5, F7, FC3, FC5, FT7), right anterior (FP2, AF4, AF8, F4, F6, F8, FC4, FC6, FT8), left posterior (CP3, CP5, TP7, P3, P5, P7, PO3, PO7, O1), and right posterior (CP4, CP6, TP8, P4, P6, P8, PO4, PO8, O2).^1^ Peak latencies were detected separately for each participant and each condition within the following time windows: 70–110 ms (N100), 180–225 ms (P200), and 225–285 ms (N200) as suggested by Luck and Kappenman (2011). A 20-ms time window was applied before and after each of the individual peaks for a mean amplitude analysis. For each time window, a repeated-measures ANOVA was calculated, using emotion (emotional, neutral), congruence (congruent, incongruent), region (anterior, posterior), and side (left, right) as within-subject factors. Only statistically significant main effects and interactions that involved the critical factors of emotion and congruence are reported in the results section. Additionally, we used the Bonferroni-Holm method to correct for multiple comparisons when looking at the interactions of side and region (Holm, 1979). Last, as a baseline calculated during the video may be affected by extraneous factors, we used the 200 ms baseline prior to the target stimulus presentation for the baseline correction.

### Results

#### Behavioral data

##### RTs

**Fig. 2:**
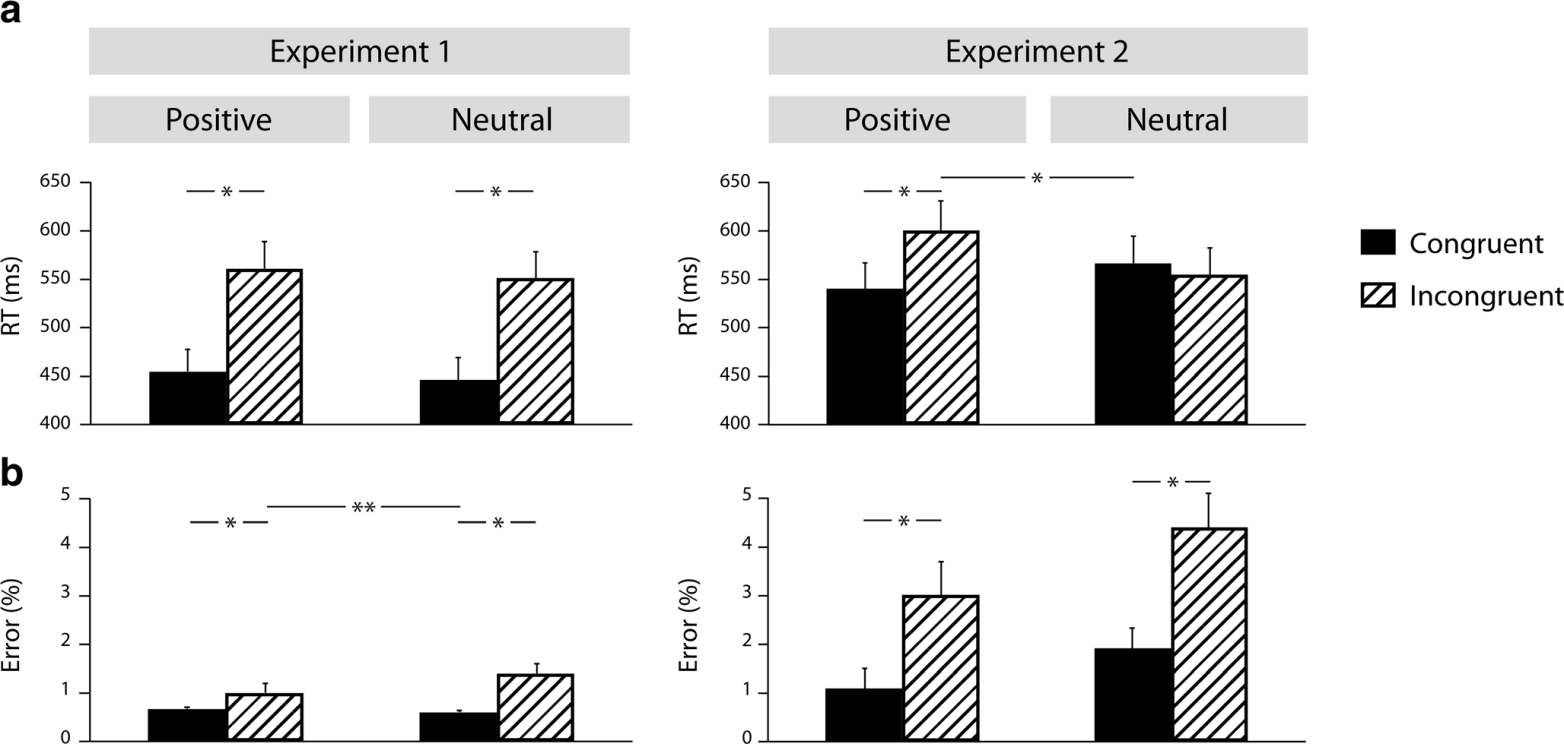
Reaction time (**a**) and error rate (**b**) data (mean + *SEM*) for congruent and incongruent/emotional and neutral conditions of Experiments 1 and 2. *Asterisk* represents the significant main effects and interactions. *Double asterisk* represents the marginally significant interaction

We observed a marginally significant main effect of emotion, F(1, 23) = 4.158, *p* = .053, *η*_*p*_^2^ = 0.153: participants responded more slowly to positive stimuli (507 ms) than to neutral stimuli (498 ms; see Fig. 2). Furthermore, incongruent videos resulted in longer RTs (556 ms) than congruent videos (450 ms), *F*(1, 23) = 353.66, *p* < .0001, *η*_*p*_^2^ = 0.939. However, the interaction of emotion by congruence was not significant, *F*(1, 23) = 0.35, *p* > .8, *η*_*p*_^2^ = 0.002.

##### Error rate

The main effect of emotion was not significant, *F*(1, 23) = 1.6, *p* > .2. Additionally, participants made more errors for incongruent (1.22%) than congruent (0.64%) stimuli, *F*(1, 23) = 8.962, *p* < .007, *η*_*p*_^2^ = 0.289. Finally, the emotion by congruence interaction showed a marginally significant effect, *F*(1, 23) = 3.194, *p* < .08, *η*_*p*_^2^ = 0.127, with a larger conflict effect (incongruent–congruent) for neutral videos (0.85%), *F*(1, 23) = 11.59, *p* < .003, *η*_*p*_^2^ = 0.345, relative to positive videos, where we observed no conflict effect, *F*(1, 23) = 2.24, *p* > .148, *η*_*p*_^2^ = 0.093.

#### ERP data

##### N100 range

**Fig. 3:**
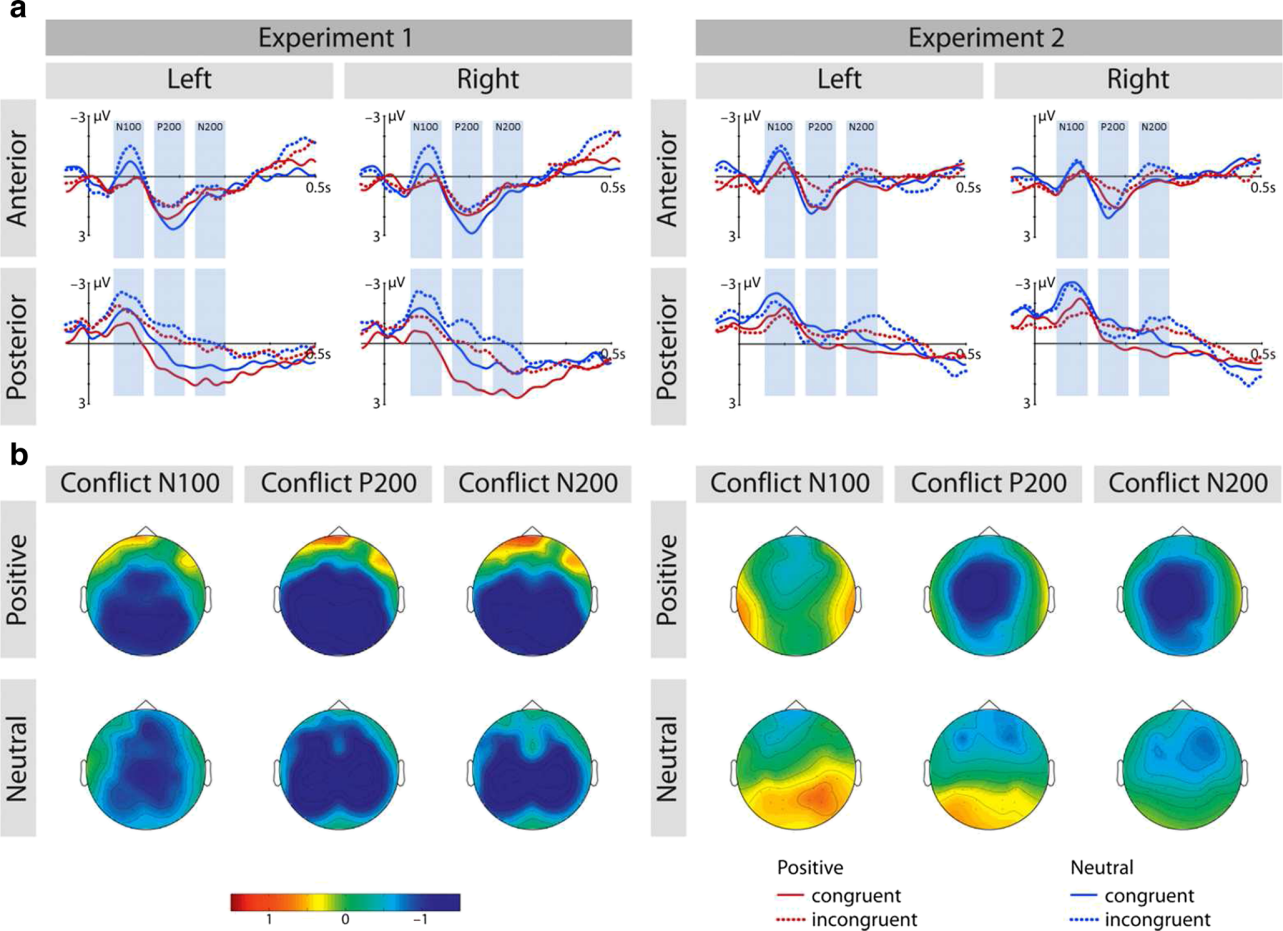
**a** ERP waves at a combination of all electrodes of interest depicting the conflict effect for positive and neutral stimuli of Experiments 1 and 2. **b** Conflict represents topographic distribution of amplitude difference (incongruent–congruent) for each of the ERP components (i.e., N100, P200, and N200 range) (Color figure online)

Neutral stimuli elicited an enhanced N1 amplitude relative to positive ones (see Fig. 3), *F*(1, 23) = 15.244, *p <* .001, *η*_*p*_^2^ = 0.399. Additionally, incongruent videos elicited an enhanced N1 amplitude compared to congruent stimuli, *F*(1, 23) = 9.969, *p* < .004, *η*_*p*_^2^ = 0.302. However, the interaction of emotion by congruence was not significant, *F*(1, 23) = 2.21, *p >* 0.1, *η*_*p*_^2^ = .088.

##### P200 range

We observed an interaction of congruence by side, *F*(1, 23) = 5.402, *p* < .029, *η*_*p*_^2^ = 0.190: Congruent stimuli led to an enhanced P200 amplitude over the left hemisphere, *F*(1, 23) = 12.091, *p* < .002, *η*_*p*_^2^ = 0.345, and a similar but stronger effect in the right hemisphere, *F*(1, 23) = 20.549, *p* < .001, *η*_*p*_^2^ = 0.472.

Additionally, we found a three-way interaction of emotion by region by side, *F*(1, 23) = 16.731, *p* < .001, *η*_*p*_^2^ = 0.421, and resolved it by side. The two-way interaction of region by emotion was significant for both left hemisphere, *F*(1, 23) = 4.63, *p* < .025, *η*_*p*_^2^ = 0.167, and right hemisphere electrode sites, *F*(1, 23) = 24.04, *p* < .001, *η*_*p*_^2^ = 0.511. We further resolved these interactions by region. Follow-up analysis showed that positive stimuli elicited an enhanced P200 amplitude relative to neutral stimuli (see Fig. 3), but only over the right posterior region, *F*(1, 23) = 6.780, *p <* .016, *η*_*p*_^2^ = 0.228. This effect was not significant in any other region (all *p*s > .1).

##### N200 range

We found an interaction of congruence by region by side, F(1, 23) = 13.759, *p* < .001, *η*_*p*_^2^ = 0.374, and resolved this interaction by side. The congruence by region interaction was significant over the right hemisphere, *F*(1, 23) = 16.38, *p* < .001, *η*_*p*_^2^ = 0.416, but not over the left hemisphere, *F*(1, 23) = 2.78, *p* > .1, *η*_*p*_^2^ = 0.108. Over the right hemisphere we further resolved this interaction by region. Over posterior brain region, incongruent videos elicited enhanced amplitudes compared to congruent videos, *F*(1, 23) = 43.99, *p* < .001, *η*_*p*_^2^ = 0.657. However, the main effect of congruence was not significant over anterior brain region, *F*(1, 23) = 0.880, *p* > .3, *η*_*p*_^2^ = 0.037.

Finally, we found a three-way interaction of emotion by region by side, *F*(1, 23) = 7.142, *p* < .014, *η*_*p*_^2^ = 0.237, and resolved it by side. The two-way interaction of emotion by region was significant over the right hemisphere, *F*(1, 23) = 5.93, *p* < .025, *η*_*p*_^2^ = 0.205, but not over the left hemisphere, *F*(1, 23) = 2.67, *p* > .1, *η*_*p*_^2^ = 0.104. In the right hemisphere, we resolved this interaction by region. Over posterior brain region, neutral videos elicited enhanced amplitudes compared to emotional videos, *F*(1, 23) = 7.519, *p* < .025, *η*_*p*_^2^ = 0.246. This effect was not significant over anterior brain region, *F*(1, 23) = 0.008, *p* > .9, *η*_*p*_^2^ = 0.0001.

To summarize, Experiment 1 tested the influence of emotional target stimuli on cognitive conflict processing with dynamic multisensory stimuli. We observed that incongruent stimuli produced overall longer RTs and higher error rates. Emotional stimuli also led to marginally longer RTs. However, positive emotions had no influence on conflict processing: The conflict effect was comparable for both positive and neutral stimuli. Further, incongruent stimuli enhanced the N100 and N200 amplitude and reduced the P200 amplitude. Neutral stimuli elicited larger ERP responses in all three ERP components. To test the influence of positive emotion of the target dimension on emotional conflict processing, we conducted Experiment 2.

## Experiment 2

### Method

#### Participants

Participants who took part in Experiment 1 also participated in Experiment 2.

#### Stimulus material and procedure

The original videos of Experiment 1 were modified to create 10 congruent and 10 incongruent videos by matching or mismatching the emotional valence of the face and voice (e.g., a facial lip movement pronouncing a neutral “A” and the corresponding audio “A” was pronounced positively; see Fig. 1). To create incongruent trials with a neutral target voice (i.e., audio trace), the original positive visual video streams were combined with neutral auditory streams. Similarly, the originally neutral visual video streams were combined with positive auditory streams to create incongruent trials with a positive target. The original voice onset was used to align the incongruent voice with the lip movement in both incongruent conditions. The vocalization of the face and voice was always matched. Participants were instructed to report whether the voice was positive or neutral.

The duration of congruent and incongruent videos ranged between 1 and 2 seconds. There were no differences between conditions in the time lag before the audio onset and total video duration (see Supplementary Material for details). The experimental design, number of trials and blocks as well as the procedure of Experiment 2 were identical to Experiment 1.

#### EEG recording and preprocessing

The EEG recording and pre-processing were identical to Experiment 1. Artifact components were removed after ICA (mean number of components removed = 17.6, *SD* = 4.7). Approximately 19% of the ERP trials (incorrect responses, artifacts) were excluded from further analysis.

#### Data analysis

Data analysis was identical to that of Experiment 1.^2^ In Experiment 2 a voice target stimulus (positive or neutral) was accompanied by either a congruent or an incongruent video.

### Results

#### Behavioral data

##### RTs

An emotion by congruence interaction was significant, *F*(1, 23) = 47.98, *p* < .001, *η*_*p*_^2^ = 0.706. The congruence effect (incongruent–congruent) was larger for positive stimuli (49 ms), *F*(1, 23) = 53.31, *p* < .002, *η*_*p*_^2^ = 0.382, than for neutral stimuli, where the conflict effect was not significant, *F*(1, 23) = 1.74, *p* > .2, *η*_*p*_^2^ = 0.080.

##### Error rate

Positive stimuli elicited fewer errors (2.05%) relative to neutral videos (3.17%), *F*(1, 23) = 4.60, *p* < .05, *η*_*p*_^2^ = 0.18. Additionally, participants produced more errors for incongruent (3.71%) compared to congruent (1.5%) stimuli, *F*(1, 23) = 35.49, *p <* .001, *η*_*p*_^2^ = 0.628. The interaction of emotion by congruence was not significant: The conflict effect was comparable across positive and neutral stimuli, *F*(1, 23) = 0.65, *p >* .4, *η*_*p*_^2^ = 0.03.

#### ERP data

##### N100 range

We found a two-way interaction of emotion by side, *F*(1, 23) = 15.227, *p* < .01, *η*_*p*_^2^ = 0.398, and resolved it by side. Neutral stimuli elicited an enhanced N100 response compared to positive stimuli, and this effect was larger over the right hemisphere, *F*(1, 23) = 16.14, *p* < .001, *η*_*p*_^2^ = 0.412, relative to the left hemisphere, *F*(1, 23) = 13.468, *p* < .001, *η*_*p*_^2^ = 0.369. However, the main effect of congruence was not significant, *F*(1, 23) = 0.44, *p* > .5, *η*_*p*_^2^ = 0.019.

##### P200 range

We found a three-way interaction of emotion by congruence by region, *F*(1, 23) = 5.629, *p* < .03, *η*_*p*_^2^ = 0.197, and resolved it by region. We further observed an interaction of emotion by congruence over the posterior electrode sites, *F*(1, 23) = 10.675, *p* < .01, *η*_*p*_^2^ = 0.317, but not over the anterior electrode sites, *F*(1, 23) = 0.383, *p* > .5, *η*_*p*_^2^ = 0.016. Over posterior sites, the main effect of congruence was significant for positive stimuli: Incongruent stimuli display a smaller amplitude than congruent stimuli, *F*(1, 23) = 8.697, *p <* .01, *η*_*p*_^2^ = 0.274. On the other hand, the congruence effect was not significant for neutral stimuli, *F*(1, 23) = 2.664, *p* > .1, *η*_*p*_^2^ = 0.104.

##### N200 range

We observed a three-way interaction of side by emotion by congruence, *F*(1, 23) = 8.067, *p* < .01, *η*_*p*_^2^ = 0.260, and resolved it by side. The two-way interaction of emotion by congruence was significant for the left hemisphere, *F*(1, 23) = 4.859, *p* < .025, *η*_*p*_^2^ = 0.146, but not for the right hemisphere, *F*(1, 23) = 0.135, *p* > .7, *η*_*p*_^2^ = 0.006. In the left hemisphere, the positive incongruent stimuli elicited a larger N200 response than congruent stimuli, *F*(1, 23) = 31.282, *p* < .001, *η*_*p*_^2^ = 0.576. This effect was not significant for neutral stimuli, *F*(1, 23) = 3.336, *p* > .05, *η*_*p*_^2^ = 0.127.

In summary, Experiment 2 tested the role of emotionality of the target dimension in emotional conflict processing. Unlike the cognitive task, emotional conflict processing resulted in a RT conflict effect for positive but not for neutral stimuli. In the ERPs, neutral stimuli enhanced N100 and P200 responses compared to positive stimuli. Furthermore, we observed a larger P200 conflict effect for positive compared to neutral stimuli over posterior electrode sites, and in the left hemisphere for the N200.

### Omnibus ANOVA

In the omnibus ANOVA we directly compared the results of Experiment 1 and Experiment 2. For each time window, a repeated-measures ANOVA was calculated using conflict type (cognitive, emotional), emotion (emotional, neutral), congruence (congruent, incongruent), region (anterior, posterior), and side (left, right) as within-subject factors.

### Results

#### Behavioral data

##### RTs

We found an interaction of conflict type by emotion by congruence, *F*(1, 23) = 26.98, *p* < .001, *η*_*p*_^2^ = 0.574, and resolved it by conflict type. Emotion influenced conflict processing in the emotional conflict task, *F*(1, 23) = 47.98, *p* < .001, *η*_*p*_^2^ = 0.706, but not in the cognitive conflict task, *F*(1, 23) = 0.35, *p* > .8, *η*_*p*_^2^ = 0.002. A follow-up analysis revealed that in the emotional conflict task the conflict effect was larger for positive stimuli (49 ms), *F*(1, 23) = 53.31, *p* < .002, *η*_*p*_^2^ = 0.382, than for neutral stimuli, *F*(1, 23) = 1.74, *p* > .2, *η*_*p*_^2^ = 0.080.

##### Error rate

Positive stimuli elicited fewer errors (1.44%) than neutral ones (2.1%), *F*(1, 23) = 5.85, *p* < .05, *η*_*p*_^2^ = 0.218. Finally, we found an interaction of conflict type by congruence, *F*(1, 23) = 12.08, *p* < .01, *η*_*p*_^2^ = 0.365, and resolved it by conflict type. Participants produced more errors for incongruent (1.22%) than for congruent (0.64%) stimuli, *F*(1, 23) = 35.49, *p <* .001, *η*_*p*_^2^ = 0.628, in the cognitive conflict task. In emotional conflict processing, the main effect of congruence was also significant (congruent = 1.51%, incongruent = 3.71%), *F*(1, 23) = 8.67, *p* < .01, *η*_*p*_^2^ = 0.292.

##### N100

We found an interaction of conflict type by congruence, *F*(1, 23) = 11.06, *p* < .01, *η*_*p*_^2^ = 0.325, and resolved it by conflict type. Incongruent compared to congruent stimuli elicited an enhanced N100 response in the cognitive conflict task, *F*(1, 23) = 9.969, *p* < .004, *η*_*p*_^2^ = 0.302, but not in the emotional conflict task, *F*(1, 23) = 0.44, *p* > .5, = 0.019. Furthermore, we found an interaction of conflict type by emotion by side, *F*(1, 23) = 9.43, *p* < .01, *η*_*p*_^2^ = 0.291. Further separate analysis for the two conflict types showed an interaction of emotion by side in the emotional conflict task, *F*(1, 23) = 15.227, *p* < .01, *η*_*p*_^2^ = 0.398, but not in the cognitive conflict task, *F*(1, 23) = 1.25, *p* > .2, *η*_*p*_^2^ = 0.052. In the emotional conflict task, neutral stimuli elicited an enhanced N100 response compared to positive stimuli, and this effect was larger over the right hemisphere, *F*(1, 23) = 16.14, *p* < .001, *η*_*p*_^2^ = 0.412, than the left hemisphere, *F*(1, 23) = 13.468, *p* < .001, *η*_*p*_^2^ = 0.369.

##### P200

We found an interaction of conflict type by emotion by congruence, *F*(1, 23) = 5.34, *p* < .030, *η*_*p*_^2^ = 0.188. Emotion influenced conflict processing in the emotional task, *F*(1, 23) = 5.25, *p* < .04, *η*_*p*_^2^ = 0.186, but not in the cognitive task, *F*(1, 23) = 1.43, *p* > .2, *η*_*p*_^2^ = 0.059. In the emotional task, the conflict effect was larger for emotional stimuli, *F*(1, 23) = 13.08, *p* < .001, *η*_*p*_^2^ = 0.363, than for neutral stimuli, *F*(1, 23) = 0.089, *p* > .7, *η*_*p*_^2^ = 0.004.

##### N200

We found an interaction of conflict type by side by emotion by congruence, *F*(1, 23) = 8.067, *p* < .01, *η*_*p*_^2^ = 0.260. The side by emotion by congruence interaction was significant in the emotional task, *F*(1, 23) = 8.067, *p* < .009, *η*_*p*_^2^ = 0.260, but not in the cognitive task, *F*(1, 23) = 1.98, *p* > 0.1, *η*_*p*_^2^ = 0.080.

In the emotional task, we resolved this interaction by side. The two-way interaction of emotion by congruence was significant for the left hemisphere, *F*(1, 23) = 4.859, *p* < .05, *η*_*p*_^2^ = 0.146, and a resolution by emotion confirmed that positive incongruent stimuli elicited a larger N200 response than congruent stimuli: *F*(1, 23) = 31.282, *p* < .001, *η*_*p*_^2^ = 0.576. This effect was not significant for neutral stimuli, *F*(1, 23) = 3.336, *p* > .05, *η*_*p*_^2^ = 0.127.

## Discussion

This study used a combined behavioral/ERP approach to study the effects of positive emotion on cognitive and emotional conflict processing. Three main findings emerged: First, an RT conflict effect was larger for positive compared to neutral stimuli in the emotional task, while positive emotions did not influence RTs in the cognitive task. Rather, positive emotions marginally reduced error rates in the cognitive task: The conflict effect was smaller for positive than for neutral stimuli. Second, the P200 and N200 responses in the emotional task showed a larger conflict effect for positive compared to neutral stimuli. In contrast, in the cognitive task we observed main effects of congruence with larger N100 and N200 responses and a smaller P200 response to incongruent than congruent stimuli. Finally, overall neutral stimuli elicited larger N100 responses than positive stimuli in both tasks.

Based on previous findings, we explored whether positive targets would facilitate cognitive conflict processing (Kanske & Kotz, 2011b; Xue et al., 2013), impede this process when the nontarget stimulus is positive (Blair et al., 2007; Dreisbach, 2006), or whether positive emotions would have no influence on cognitive conflict processing (Martin & Kerns, 2011). In line with some of this previous evidence, the RT results of Experiment 1 show that positive emotion does not influence cognitive conflict processing (Martin & Kerns, 2011), while overall RTs were marginally prolonged for positive compared to neutral stimuli (Blair et al., 2007). Possibly, as the cognitive conflict (Experiment 1) was easy (error rate ~1%), performance was already at ceiling, and positive emotions could not speed up conflict processing (or influence it in any other way). Previous studies have demonstrated enhanced accuracy and shorter RTs for the processing of multisensory emotional information relative to unimodal emotional information (De Gelder & Vroomen, 2000; Kreifelts, Ethofer, Grodd, Erb, & Wildgruber, 2007). In other words, due to the salient multisensory nature of the stimuli used in the current set of experiments, the cognitive conflict task may not have been demanding enough, and the influence of positive emotions was thus minimized.

In line with our predictions, we observed a conflict-related N200 (Bruin & Wijers, 2002). The N200 response was enhanced for incongruent relative to congruent trials. This is in line with previous unimodal studies that proposed that the N200 is a conflict-sensitive ERP component (Kopp et al., 1996; van Veen & Carter, 2002). Also, as hypothesized, dynamic multisensory stimuli receive prioritized perceptual processing relative to unimodal stimuli (Franconeri & Simons, 2003) and lead to an early modulation of conflict-sensitive ERP components (Jessen & Kotz, 2011; Paulmann et al., 2009; Zinchenko et al., 2015). For instance, we observed a conflict effect in the P200 range: Incongruent P200 responses were smaller than congruent P200 responses (Kokinous et al., 2014). Increased allocation of attention has been linked to a decrease in the P200 amplitude (Crowley & Colrain, 2004). Therefore, we interpret the current P200 results as induced by a greater attentional demand and less attentional allocation to the vocalization when the task-irrelevant face was incongruent. Finally, the N100 was sensitive to congruence. This finding implies that cognitive conflict can already be detected as early as 100 ms after stimulus onset when using dynamic multisensory stimuli (Zinchenko et al., 2015).

Unexpectedly, in the emotional task the behavioral conflict effect was larger for positive than for neutral stimuli. Even so, this result is in line with several previous studies that showed positive emotion to increase distractibility (Dreisbach & Goschke, 2004) and to reduce the use of informative cues (Froeber & Dreisbach, 2012). Visual information typically and naturally precedes auditory information (Chandrasekaran, Trubanova, Stillittano, Caplier, & Ghazanfar, 2009; Jessen & Kotz, 2013; Stekelenburg & Vroomen, 2012). Moreover, preceding [affective] visual information primes acoustic information, facilitates the processing of the auditory modality (e.g., Besle, Fort, Delpuech, & Giard, 2004; Klucharev, Mottonen, & Sams, 2003; van Wassenhove, Grant, & Poeppel, 2005), and elicits stronger reactions than the auditory modality (for a review, see Gerdes, Wieser, & Alpers, 2014). Dreisbach (2006) showed that positive compared to neutral emotional pictures presented before each trial reduce the prediction strength between the cue and a subsequent target. Hence, when a facial cue in an emotional task is positive (i.e., in the neutral incongruent condition), it forms weaker predictions about the upcoming target. This weaker prediction becomes beneficial when the subsequent target is incongruent with the preceding cue, as people can easily overcome and inhibit prepotent response tendencies. Therefore, we observed the “surprising” absence of a conflict effect in the neutral target condition, while positive target stimuli showed a conventional conflict effect. On the other hand, the analysis of error rates revealed that positive face cues could still create a propensity to misidentify the neutral voices as positive, despite the absence of a RT conflict effect. This is not consistent with the findings of Dreisbach (2006), who showed reduced error rates for positive compared to neutral emotions. This could be caused by the robust natural links between affective face and voice in the current task, which, when not matching, could result in conflict. Alternatively, Dreisbach (2006) used completely task-irrelevant affective pictures that preceded each trial in the AX-CPT task. Moreover, while the cue itself served as a distractor in our task, in Dreisbach’s (2006) study these were different and unrelated objects. Therefore, we conclude that positive cues reduced the prediction strength in the emotional conflict task, but the conflict was not always avoidable due to the strong natural relationship between faces and voices. Finally, positive emotion did not influence the cognitive task, possibly because the expressed emotion of the target (i.e., voice) was always congruent with and correctly predicted by a preceding face.

Goal-directed behavior in a dynamic environment requires the ability to maintain intentions and goals over time. However, the ability to maintain a goal may also be detrimental, when a prediction is wrong and the goal has to be flexibly adjusted. Our findings indicate that positive emotions may reduce the ability to maintain a goal and thus promote flexibility (Dreisbach, 2006).

In the ERPs, we observed that positive rather than neutral emotions enhanced the N200 conflict effect (van Veen & Carter, 2002; Zinchenko et al., 2015). This is in line with the behavioral findings and indicates that increased neural resources are required to overcome conflict in the positive relative to the neutral target condition. Furthermore, the N200 is also enhanced when an incorrect response is suppressed (Azizian, Freitas, Parvaz, & Squires, 2006; Luck, 2005). Therefore, it was more difficult for participants to suppress incorrect responses when the distractor face was neutral rather than positive.

Furthermore, we found that the conflict effect was larger for the positive stimuli relative to neutral stimuli in the P200 (Ho et al., 2014; Kokinous et al. 2014; Zinchenko et al., 2015). As stated above, the decreased P200 amplitude has been linked to an increase in the allocation of attention (Crowley & Colrain, 2004). Therefore, we interpret the current P200 effect similarly—there was less attentional allocation to the vocalization when the distractor was neutral than when it was positive. These results also go hand in hand with a recent study on multisensory emotional audiovisual integration that reported a decrease in the P200 amplitude for incongruent stimuli, which reflects reduced information gain from incongruent stimuli (Kokinous et al., 2014). Therefore, our findings indicate that incongruent trials are more demanding when the distractor is neutral than when it is positive, possibly because positive emotions lead to the reduced use of informative cues (Froeber & Dreisbach, 2012). Finally, Knowland and colleagues (2014) suggested that the audiovisual P200 suppression reflects competition between different multisensory inputs with greater competition for incompatible stimulation. This implies once again that there may be less attentional competition when distracting multisensory input is positive rather than neutral.

In contrast to the cognitive task, we found no main effect of congruence in the N100 in the emotional task. Nevertheless, target emotionality influenced the N100, as neutral trials elicited a larger N100 than positive emotion trials in both tasks. We interpret this finding as a reduced use of informative cues (Froeber & Dreisbach, 2012) as well as an increase in the breadth of attention in the positive relative to neutral emotions (Rowe et al., 2007). In line with this hypothesis, Gable and Harmon-Jones (2012) showed that increasing the breadth of attention by manipulating the global attentional scope attenuates the attentional capture of emotional stimuli and reduces the N100 amplitude.

Furthermore, our findings suggest that the emotional quality of the target stimulus influences conflict-related facilitation of attention in the emotional but not in the cognitive conflict task. This dissociation is particularly striking, as we used identical visual and auditory stimuli in the emotional and cognitive tasks. In general, the influence of emotion on different types of conflict processing seems to be highly interconnected with a number of attentional processes. Emotion can facilitate, inhibit, or have no influence on executive function dependent on its task relevance as well as its ability to either attract or inhibit allocation of attention.

Previous studies reported inconsistent results regarding the distribution of N100, P200, and N200 conflict effects. For instance, the N100 conflict effect was previously observed over anterior regions (Kokinous et al., 2015; Liu et al., 2012), central regions (Pourtois, Debatisse, Despland, & de Gelder, 2002), or over posterior regions only (Ho et al., 2015). The P200 conflict effect was shown over anterior regions (Ho et al., 2015; Kokinous et al., 2015), but also over posterior regions only (Gerdes et al., 2013). Similarly, several studies found the N200 conflict effect at frontocentral electrodes (Heil et al., 2000; Kopp et al., 1996; Kanske & Kotz, 2011), and over posterior regions (Hughes et al., 2009; Zinchenko et al., 2015). In a similar vein, we found an interaction of emotion by conflict across all regions (e.g., N200, emotional task), and in the left hemisphere only (e.g., N200, cognitive task). Therefore, further studies will need to address the question of the distribution of conflict-specific ERP components more systematically.

The behavioral and ERP results regarding the influence of positive emotion on the two conflict types are in contrast to our previous results on negative emotions (Zinchenko et al., 2015) as negative emotion facilitated conflict processing in both tasks. We interpret this behavioral discrepancy as reflecting valence-specific mechanisms underlying conflict-specific tasks (Ochsner, Hughes, Robertson, Cooper, & Gabrieli, 2009; Soutschek & Schubert, 2013). More specifically, cognitive conflict is resolved by amplified processing of the target stimulus (Egner & Hirsch, 2005), while emotional conflict processing seems to lead to facilitated inhibition of the non-target distractor dimension of a stimulus (Etkin et al., 2006). As negative emotion seems to narrow the breadth of attention (see Eysenck et al., 2007, for a review), it facilitates conflict processing in both cognitive and emotional conflict tasks by either amplifying the processing of the target dimension (cognitive conflict), or reducing the influence from the task-irrelevant emotional distractor (emotional conflict; Zinchenko et al., 2015). On the other hand, positive emotions appear to broaden the scope of visual attention (e.g., Johnson et al., 2010) and either have a negligible influence on cognitive control or reduce the conflict effect in the emotional conflict task. In other words, our study showed that a dissociation of cognitive and emotional control mechanisms is complex and modulated by valence. It seems that conflict-specific processing is not only dependent on the conflict type, but also on the emotionality of the target dimension as observed in the differential behavioral and neural responses for neutral and emotional targets within both cognitive and emotional tasks.

Additionally, Kanske and Kotz (2012) showed that the influence of positive emotions on conflict processing correlates with interpersonal characteristics, such as effortful control, anxiety, and depression. Therefore, further studies will have to take into consideration interpersonal characteristics in order to further clarify the role of positive emotion in cognitive and emotional conflict processing.

Finally, in contrast to the within-trial conflict processing analyzed in the current set of experiments, cognitive and emotional control have also been investigated using the conflict adaptation setup, that is, conflict processing as a function of congruence of the previous trial (congruent, incongruent; e.g., Alguacil et al., 2013; van Steenbergen, Band, & Hommel, 2010). As the current set of experiments was designed to focus on the influence of emotion (and not the previous-trial congruence) on the within-trial conflict processing, the low number of repetitions in the current experimental setup makes it impossible to further explore conflict adaptation effects. However, it is important to note that between trial conflict adaptation may also influence current trial conflict effects. Therefore, future research should study how the emotional quality of the target influences cognitive and emotional conflict adaptation using dynamic multisensory stimuli.

### Conclusion

One of the major requirements of successful social interaction is the ability to process complex multimodal information that is derived from distinct sensory inputs. This becomes particularly difficult when distinct emotional inputs are in conflict with each other. In two ERP experiments we have shown that positive target stimuli have no influence on cognitive conflict processing, but impede emotional conflict processing. In line with behavioral findings, we found corresponding effects in the N100, P200, and N200 components. Our results suggest that there are distinct neuropsychological mechanisms for processing cognitive and emotional tasks, which seem to vary as a function of emotional valence.

## Electronic supplementary material

Below is the link to the electronic supplementary material.

ESM 1 (DOCX 32 kb)

## Footnotes

1 Conflict processing has repeatedly been observed for the N200 component over anterior midline electrodes. Please see the Supplementary Material for an additional analysis of midline electrode sites for the N200 component in both Experiments. These data were identical to the results of analyses of lateral electrode sites.

2 Please find the results of an Omnibus ANOVA that compares data across both Experiments 1 and 2, adding the within-group factor of conflict type (cognitive, emotional) at the end of the results section.
