# Supplementary material for: Positive emotion impedes emotional but not cognitive conflict processing
Source: Cogn Affect Behav Neurosci. 2017 Mar 20;17(3):665–77. doi: 10.3758/s13415-017-0504-1 (PMC5403863; doi:10.3758/s13415-017-0504-1)
Supplement: Supplementary file 1 — (DOCX 32 kb) [file 13415_2017_504_MOESM1_ESM.docx]

Supplemental Materials

**Experiment 1**

**Timing**

There was no significant delay difference between the start of the video and the voice onset of videos between vocalization (i.e., “A” and “O”, paired-t(7) = -1.98, *p* > 0.09, *d* = -0.115), emotion (i.e., neutral and emotional, paired-t(7) = -1.24, *p* > 0.26, *d* = -0.072), and congruence (i.e., congruent, incongruent, paired-t(7) = 0.01, *p* > 0.4, *d* = 0.001) (see Table 1). For the total video duration, there was no significant difference between vocalization (paired-t(7) = -1.86, *p* > 0.1, *d* = -0.067) and congruence (paired-t(7) = 0.58, *p* > 0.5, *d* = 0.021). The total video duration was longer for positive compared to neutral videos (paired-t(7) = -3.22, *p* < 0.05, *d* = -0.116).

**Ratings**

Complete videos, video streams alone, and audio streams alone were rated on a 7-point Likert scale using Self-Assessment Manikins in terms of expressiveness, arousal and emotion identification (Bradley & Lang, 1994) by 24 (12 female) participants (see Table 2). A paired-samples t-test for the complete videos showed no main effect of emotion for either arousal (paired-t(23) = -0.105, *p > 0*.9, *d* = -0.066 ) or for expressiveness (paired-t(23) = -0.424, *p >* 0.6, *d* = -0.269). The main effect of emotion was significant in the ratings of valence: participants rated emotional videos as more emotional compared to neutral videos (paired-t(23) = 10.04, *p* < 0.01, *d* = 8.061). For the audio streams alone, the result of the analysis showed no main effect of emotion for either arousal (paired-t(23) = 2.78, *p >* 0.7, *d* = 1.898) or for expressiveness (paired-t(23) = -4.11, *p >* 0.6, *d* = -2.820). However, the main effect of emotion was significant for the ratings of valence (paired-t(23) = 7.62, *p <* 0.01, *d* = 5.591): emotional videos were rated as more emotional relative to neutral videos. For the video stream alone, there was no main effect of emotion for either arousal (paired-t(23) = 0.385, *p >* 0.7, *d* = 0.253) or for expressiveness (paired-t(23) = 1.53, *p >* 0.1, *d* = 0.172). The main effect of valence was significant (paired-t(23) = 10.47, *p <* 0.01, *d* = 0.125).

**Midline electrodes** (anterior midline: FPZ, AFZ, FZ, FCZ, AZ; posterior midline: CPZ, PZ, POZ, OZ, PZ).

**N200**

We found an interaction of emotion by region. Neutral stimuli elicited enhanced amplitudes relative to positive stimuli over posterior brain region (F(1, 23) = 9.10, p < 0.01, = 0.283). This effect was not significant over anterior brain region (F(1, 23) = 0.017, *p* > 0.8, = 0.001). The main effect of congruence was significant: incongruent stimuli elicited enhanced amplitude compared to congruent stimuli (F(1, 23) = 21.022, *p* < 0.001, = 0.478). Furthermore, we observed a significant interaction of congruence by region (F(1, 23) = 18.29, *p* < 0.001, = 0.443). We resolved this interaction by region. In the posterior region, incongruent stimuli elicited enhanced amplitude compared to congruent stimuli (F(1, 23) = 46.56, *p* < 0.001, = 0.669). The main effect of congruence was not significant over the anterior brain region (F(1, 23) = 3.25, *p* > 0.05, = 0.124).

**Experiment 2**

Timing

There was no significant delay between the start of the video and the voice onset of videos between vocalization (i.e., “A” and “O”, paired-t(7) = 1.49, *p* > 0.1, *d* = 0.855), emotion (i.e., neutral and emotional, paired-t(7) = 0.211, *p* > 0.8, *d* = 0.012), and congruence (i.e., congruent, incongruent, paired-t(7) = 0.420, *p* > 0.6, *d* = 0.001) (see Table 3). For the total video duration, there was no significant difference between vocalization (paired-t(7) = 1.54, *p* > 0.1, *d* = 0.055), congruence (paired-t(7) = 0.0, *p* = 1) and emotion (paired-t(7) = 0.0, *p* = 1).

**Midline electrodes** (anterior midline: FPZ, AFZ, FZ, FCZ, AZ; posterior midline: CPZ, PZ, POZ, OZ, PZ).

**N200**

The main effect of emotion was significant (F(1, 23) = 10.19, *p* < 0.004, = 0.307). Furthermore, we also found an interaction of emotion by region (F(1, 23) = 4.24, *p* < 0.05, = 0.156). In the posterior brain region, positive stimuli elicited larger responses than neutral stimuli (F(1, 23) = 15.49, *p* < 0.001, = 0.513). This effect was not significant over anterior brain regions (F(1, 23) = 2.53, *p* > 0.1, = 0.099). Finally, we also found a significant main effect of congruence (F(1, 23) = 26.46, *p* < 0.001, = 0.535). Incongruent stimuli elicited enhanced amplitude compared to congruent stimuli.

**Movement**

To check whether the videos differed with regards to movement, we estimated the number of movements per video clip by quantifying the variation of light intensity (luminance) between pairs of frames for each pixel (Pichon et al., 2008). The two emotions (neutral and emotional) and two vowels (‘A’ and ‘O’) were compared with a Kruskal-Wallis test. The motion difference was not significant between positive and neutral videos (X² = 3.00, *p* > 0.05) and between vowels (X² = 1.25, *p* > 0.2).

Table 1

*Timing of video stimuli of Experiment 1*

| Video condition (the “vowel” specifies the interjection) | Time before start of the movement (ms) | Time before start of the audio sound (ms) | Total video duration (ms) |
| --- | --- | --- | --- |
| **Female** |  |  |  |
| Neutral congruent “A” | 240 | 561 | 1400 |
| Neutral congruent “O” | 240 | 740 | 1480 |
| Positive congruent “A” | 240 | 600 | 1680 |
| Positive congruent “O” | 240 | 951 | 1960 |
| Face Neutral “A” - Voice Neutral “O” | 240 | 540 | 1400 |
| Face Neutral “O” - Voice Neutral “A” | 240 | 562 | 1480 |
| Face Positive “A” - Voice Positive “O” | 240 | 472 | 1680 |
| Face Positive “O” - Voice Positive “A” | 240 | 760 | 1960 |
| **Male** |  |  |  |
| Neutral congruent “A” | 240 | 475 | 1400 |
| Neutral congruent “O” | 240 | 560 | 1400 |
| Positive congruent “A” | 240 | 420 | 1400 |
| Positive congruent “O” | 240 | 760 | 1720 |
| Face Neutral “A” - Voice Neutral “O” | 240 | 520 | 1400 |
| Face Neutral “O” - Voice Neutral “A” | 240 | 635 | 1400 |
| Face Positive “A” - Voice Positive “O” | 240 | 440 | 1400 |
| Face Positive “O” - Voice Positive “A” | 240 | 540 | 1640 |

Table 2

*Results of the video rating*

| Stimuli |  | Arousal | Expressiveness | Valence |
| --- | --- | --- | --- | --- |
| Complete video | Neutral | 4.97 (2.92) | 4.80 (2.08) | 5.38 (0.45) |
|  | Positive | 5.07 (1.84) | 5.16 (2.39) | 2.23 (1.46) |
| Audio stream | Neutral | 4.23 (1.18) | 4.29 (1.08) | 4.96 (1.25) |
|  | Positive | 4.35 (0.73) | 4.21 (0.95) | 2.97 (0.19) |
| Videos stream | Neutral | 4.37 (1.45) | 4.63 (1.46) | 4.91 (0.93) |
|  | Positive | 4.60 (1.20) | 4.14 (0.95) | 3.05 (0.94) |

*Note.* Arousal and expressiveness were rated on a scale from 1 (low) to 9 (high). Valence of videos was rated on a scale from 1 (positive) to 9 (negative). Values represent mean and standard deviation.

Table 3

*Timing of video stimuli of Experiment 2*

| Video condition (the “vowel” specifies the interjection) | Time before start of the movement (ms) | Time before start of the audio sound (ms) | Total video duration (ms) |
| --- | --- | --- | --- |
| **Female** |  |  |  |
| Neutral congruent “A” | 240 | 561 | 1400 |
| Neutral congruent “O” | 240 | 740 | 1480 |
| Positive congruent “A” | 240 | 600 | 1680 |
| Positive congruent “O” | 240 | 951 | 1960 |
| Face Neutral - Voice Positive “A” | 240 | 640 | 1400 |
| Face Neutral - Voice Positive “O” | 240 | 550 | 1480 |
| Face Positive - Voice Neutral “A” | 240 | 560 | 1680 |
| Face Positive - Voice Neutral “O” | 240 | 776 | 1960 |
| **Male** |  |  |  |
| Neutral congruent “A” | 240 | 475 | 1400 |
| Neutral congruent “O” | 240 | 560 | 1400 |
| Positive congruent “A” | 240 | 420 | 1400 |
| Positive congruent “O” | 240 | 760 | 1720 |
| Face Neutral - Voice Positive “A” | 240 | 320 | 1400 |
| Face Neutral - Voice Positive “O” | 240 | 760 | 1400 |
| Face Positive - Voice Neutral “A” | 240 | 634 | 1400 |
| Face Positive - Voice Neutral “O” | 240 | 560 | 1720 |

References:

Pichon, S., de Gelder, B., Grezes, J. (2008). Emotional modulation of visual and motor areas by dynamic body expressions of anger. *Social Neuroscience*, 3(3–4), 199–212.
